# Association of common gene variants in glucokinase regulatory protein with cardiorenal disease: A systematic review and meta-analysis

**DOI:** 10.1371/journal.pone.0206174

**Published:** 2018-10-23

**Authors:** Pomme I. H. G. Simons, Nynke Simons, Coen D. A. Stehouwer, Casper G. Schalkwijk, Nicolaas C. Schaper, Martijn C. G. J. Brouwers

**Affiliations:** 1 Department of Internal Medicine, Division of Endocrinology, Maastricht University Medical Center, Maastricht, The Netherlands; 2 Department of Internal Medicine, Division of General Internal Medicine, Laboratory for Metabolism and Vascular Medicine, Maastricht University Medical Center, Maastricht, The Netherlands; 3 CARIM, School for Cardiovascular Diseases, Maastricht, The Netherlands; 4 Department of Internal Medicine, Division of General Internal Medicine, Maastricht University Medical Center, Maastricht, The Netherlands; 5 School for Public Health and Primary Care (CAPHRI), Maastricht, The Netherlands; University of Mississippi Medical Center, UNITED STATES

## Abstract

**Background:**

Small-molecules that disrupt the binding between glucokinase and glucokinase regulatory protein (GKRP) in the liver represent a potential new class of glucose-lowering drugs. It will, however, take years before their effects on clinically relevant cardiovascular endpoints are known. The purpose of this study was to estimate the effects of these drugs on cardiorenal outcomes by studying variants in the GKRP gene (*GCKR*) that mimic glucokinase-GKRP disruptors.

**Methods:**

The MEDLINE and EMBASE databases were searched for studies reporting on the association between *GCKR* variants (rs1260326, rs780094, and rs780093) and coronary artery disease (CAD), estimated glomerular filtration rate (eGFR), and chronic kidney disease (CKD).

**Results:**

In total 5 CAD studies (n = 274,625 individuals), 7 eGFR studies (n = 195,195 individuals), and 4 CKD studies (n = 31,642 cases and n = 408,432 controls) were included. Meta-analysis revealed a significant association between *GCKR* variants and CAD (OR:1.02 per risk allele, 95%CI:1.00–1.04, p = 0.01). Sensitivity analyses showed that replacement of one large, influential CAD study by two other, partly overlapping studies resulted in similar point estimates, albeit less precise (OR:1.02; 95%CI:0.98–1.06 and OR: 1.02; 95%CI: 0.99–1.04). *GCKR* was associated with an improved eGFR (+0.49 ml/min, 95%CI:0.10–0.89, p = 0.01) and a trend towards protection from CKD (OR:0.98, 95%CI:0.95–1.01, p = 0.13).

**Conclusion:**

This study suggests that increased glucokinase-GKRP disruption has beneficial effects on eGFR, but these may be offset by a disadvantageous effect on coronary artery disease risk. Further studies are warranted to elucidate the mechanistic link between hepatic glucose metabolism and eGFR.

## Background

In the current area of precise medicine, there is an ongoing search for new anti-diabetic medication with different modes of action. Drugs that modulate the function of glucokinase have been the scope of diabetes research for more than a decade now [1–4]. Glucokinase plays a pivotal role in regulating pancreatic insulin secretion and hepatic glucose uptake, owing to its unique enzymatic actions [5]. It catalyzes the conversion of glucose to glucose-6-phosphate, the first step in glycolysis. To date, however, clinical trials with glucokinase activators in patients with type 2 diabetes have been disappointing, since the glucose-lowering effects were non-sustained and accompanied by an increased risk of hypoglycemia and hypertriglyceridemia [1]. Hepatoselective glucokinase activators could theoretically bypass some of these side-effects, in particular the risk of hypoglycemia [6].

An alternative way to increase hepatic glucokinase activity is to disrupt the binding between glucokinase and glucokinase regulatory protein (GKRP). GKRP is a liver-specific protein located in the nucleus that binds–and hence inactivates–glucokinase in the fasting state. In the postprandial state, glucokinase dissociates from GKRP and subsequently migrates towards the cytosolic space where it facilitates phosphorylation of glucose [7, 8]. Lloyd and colleagues previously demonstrated that small molecules that disrupt the glucokinase-GKRP complex reduce plasma glucose levels without causing hypoglycemia in mice [9]. Although promising, it will probably take years before this new drug can be tested in a clinical setting.

Genetic epidemiology can be helpful to gain more insight into the clinical effects of glucokinase-GKRP disruption in humans. Since individuals are ‘randomized’ at birth to receive a wildtype allele or a variant that encodes GKRP that binds glucokinase less effectively, the effects of this variant on clinical endpoints can be studied as a surrogate for glucokinase-GKRP disruptors. Such a Mendelian randomization approach has been proven to be effective in predicting the (un)intended effects of new drugs [10].

We previously reviewed current literature on the cardiometabolic effects of variants in the glucokinase regulatory protein gene (*GCKR*) [11]. Individuals carrying the variant that binds glucokinase less effectively are indeed characterized by reduced fasting plasma glucose levels, but this is accompanied by an increased risk of nonalcoholic fatty liver disease (NAFLD), hypertriglyceridemia, and gout [12–14]. Of interest, there are studies suggesting that the same variant protects from chronic kidney disease (CKD) [15]. Given these opposing effects it is difficult to predict what the net effect will be on coronary artery disease (CAD), one of the most clinically relevant outcomes in type 2 diabetes.

The aim of the present study was therefore to elucidate the association between *GCKR* and CAD and CKD by conducting a systematic review and meta-analysis.

## Methods

### Data sources, searches, and study selection

The MEDLINE and EMBASE databases were searched for: 1) original, genetic association studies addressing the relationship between common variants in *GCKR* (rs1260326, rs780094, or rs780093) and CAD; and 2) genome-wide association studies (GWAS) on CAD, as they are likely to include the variants of interest (see S1 Table for search strategy and S1 Fig for flowchart). CAD was defined as myocardial infarction (MI), significant stenosis (i.e. ≥50%) in one or more main coronary arteries, or coronary intervention, including coronary artery bypass grafting (CABG) and percutaneous coronary intervention (PCI).

A second search was performed for the association between the common variants in *GCKR* and renal function. Studies reporting serum creatinine levels, eGFR (based on serum creatinine or cystatin C), or presence of CKD were considered eligible (see S2 Table for search strategy and S2 Fig for flowchart).

Cross-sectional articles, written in English, German, or Dutch, were included. No publication date or publication status restrictions were imposed. The electronic searches were conducted by one researcher (P.I.H.G.S.) and completed on March 6, 2018.

### Meta-analyses

Two separate systematic reviews and three meta-analyses were conducted to determine the association between 1) common variants in *GCKR* and CAD; and 2) common variants in *GCKR* and renal function, i.e. estimated glomerular filtration (eGFR) and chronic kidney disease (CKD; based on dichotomized eGFR). Selection of variants was primarily based on functionality, i.e. the variant has been shown to be functional and mimics the effects of glucokinase-GKRP disruptors (i.e. rs1260326 [16, 17]). In addition, variants that are in strong linkage disequilibrium with this functional variant, i.e. rs780094 or rs780093, were included as well (r^2^ ≈ 0.92 for both SNPs in both Europeans and East Asians; source: 1000 Genomes project phase 3). The systematic reviews and meta-analyses were performed according to the PRISMA statement with the only exception of a (registered) review protocol (see S1 Checklist) [18].

### Data extraction and quality assessment

Data extraction was done in a two-step, standardized fashion where one researcher (P.I.H.G.S.) extracted the data, which was subsequently checked by two other researchers (N.S. and M.C.G.J.B.). The following variables were extracted from the included studies: odds ratios or unstandardized beta coefficients, with 95% confidence intervals or standard errors. Authors were contacted in case of missing data (in particular for the GWAS). In case of non-response, a reminder was sent three weeks later. When more than one *GCKR* variant was reported, the functional variant (rs1260326) was chosen. The additive model was the preferred model of inheritance, based on previous *GCKR* association studies [19]. Finally, given our interest in the systematic effects of *GCKR* per se, we aimed to obtain the crude outcome variables, i.e. without adjustment for potential mediators (e.g. plasma lipids levels).

To avoid inclusion of study cohorts that were reported more than once, in particular in GWAS consortia, special attention was paid to the origin of the individual study populations. In case of overlap, the study that contained the highest number of participants was included. The quality of the study and the risk of bias were assessed by two independent researchers (P.I.H.G.S. and N.S.) according to the Newcastle-Ottawa Scale (NOS) [20].

### Data synthesis and analysis

Back-transformation of the log-transformed difference in eGFR between the two *GCKR* alleles was done as described elsewhere [21]. Odds ratios and beta coefficients were meta-analyzed based on a random-effects model, using the DerSimonian-Laird method to incorporate between-study heterogeneity. Funnel plots were visually examined for asymmetry and analyzed by means of regression (Egger’s test).

Since most studies (in particular GWAS) only reported the principal summary measures (i.e. odds ratios) instead of individualized data, it was not feasible to adjust for potential environmental effects, nor was it possible to assess Hardy-Weinberg equilibrium or linkage disequilibrium for each study.

Sensitivity analyses were performed to assess the impact of studies that included subjects with different ancestries, studies with low quality (defined as a NOS score <5 stars), and studies that did not report crude (or age- and/or sex-adjusted) estimates. All analyses were conducted with the ‘R’ statistical software (R Developmental Core Team) using the *metaphor* package [22].

## Results

### Systematic review and meta-analysis of the association between common variants in *GCKR* and CAD

The electronic search identified 3,051 unique records, which eventually resulted in five studies that were used for the meta-analysis [23–27] (see S1 Fig for flowchart and reasons for exclusion). All included studies were written in English. Twenty-six studies showed overlap with one of the included studies, i.e. the combined UK Biobank, CARDIoGRAMplusC4D 1000 genomes-based GWAS, and Myocardial Infarction Genetics and CARDIoGRAM Exome dataset [24], and were therefore not included in the meta-analysis (S3 Table). The genetic variants of interest were often not reported in the main article, but could be found in the (online) supplementary materials of the article. For one GWAS, the authors were contacted and the requested data were kindly provided [25].

The characteristics of the included studies are shown in Table 1. In total, 274,625 subjects were included. In some, mainly Asian studies, the *GCKR* effect allele–defined as the allele that predisposes to reduced fasting plasma glucose levels (similar to the effect of a glucokinase-GKRP disruptor)–was the predominant allele. The overall quality of the studies was good (S4 Table).

**Table 1:** Characteristics of included studies on coronary artery disease (CAD).

Author	Year	Ancestry	Population type	Number of cases	Number of controls	Covariates adjusted for	SNP	EAF	Outcome
**Lian [23]**	2013	Asian	Hospital	568	494	-	rs780093	0.52	CHD
**Nelson [24]**	2017	European + non-European	General + hospital	268,744*	-	Array and population structure/ancestry	rs1260326	0.40	CAD
**Raffield [25]**	2015	European	Type 2 diabetes	212	771	Age, sex	rs1260326	0.39	MI
**Takeuchi [26]**	2012	Asian	Hospital	1,347	1,337	Not specified	rs780094	0.56	CAD
**Zhou [27]**	2015	Asian	General + hospital	555	597	-	rs1260326	0.42	CAD

*Number of cases refers to the overall population.

Abbreviations: SNP: single nucleotide polymorphism; EAF: effect allele frequency; CHD: coronary heart disease; MI: myocardial infarction.

**Fig 1:**
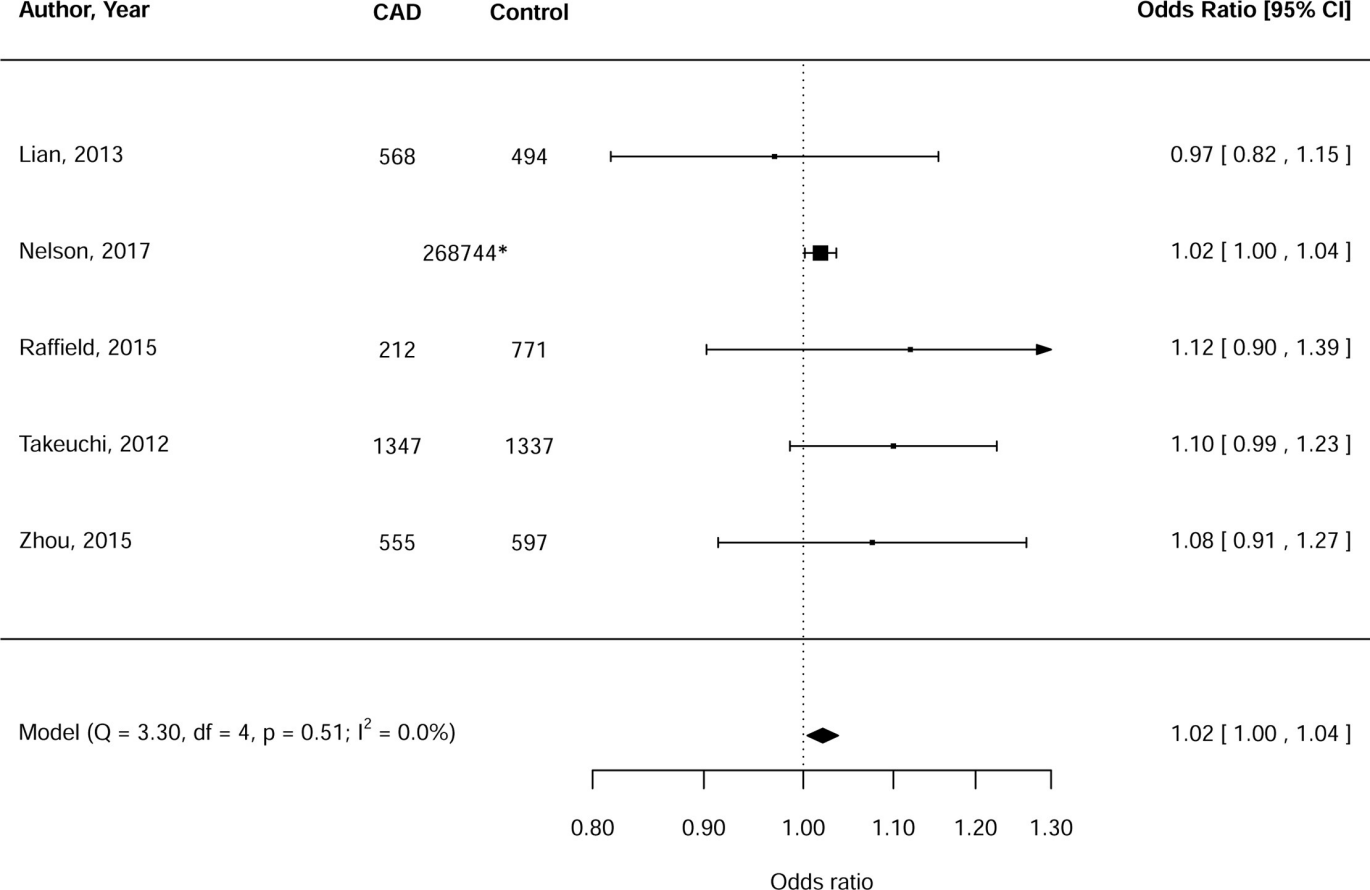
Meta-analysis of the relationship between the *GCKR* effect allele and coronary artery disease (CAD). *Number of individuals refers to the overall population.

Meta-analysis demonstrated that the *GCKR* effect allele was significantly associated with CAD (OR: 1.02, 95%CI: 1.00–1.04, p = 0.01) (Fig 1). Heterogeneity was low (Q = 3.30, I^2^ = 0%) [28]. Due to the low number of included studies, a funnel plot (or testing for funnel plot asymmetry) was not included, according to previous recommendations [29, 30]. Since the meta-analysis was dominated by one large study–which is composed of 76 sub-studies [31]–we conducted several sensitivity analyses to test the robustness of our findings. First, this large study was replaced by another large study that combined the CARDIoGRAMplusC4D 1000 genomes-based GWAS dataset with an additional 56,354 samples (n = 260,365 subjects in total, S3 Table) [32]. The subsequent meta-analysis revealed a similar, but less precise point estimate (OR: 1.02, 95%CI: 0.98–1.06, p = 0.37, S3 Fig). The initial large study was also replaced by the CARDIoGRAMplusC4D Metabochip data [33, 34], which overlaps for ~55% with the CARDIoGRAMplusC4D 1000 genomes-based GWAS data (S3 Table) [35]. This also allowed a better sensitivity analysis stratified by ancestry, since data for Europeans only have been presented separately [34]. Again, the overall meta-analysis showed a similar, but non-significant point estimate (OR: 1.02, 95%CI: 0.99–1.05, p = 0.27, S4 Fig).

The *GCKR* effect allele was significantly associated with CAD in studies that included subjects of European ancestry only (n = 3) (OR: 1.02, 95%CI: 1.00–1.05, p = 0.02), but not in studies that included subjects of Asian ancestry only (OR: 1.06, 95%CI: 0.98–1.15, p = 0.13; S4 Fig). Of note, these effect sizes were not statistically different (p = 0.36). Repeat analysis without the study with low quality [25] (i.e. NOS score <5 stars) did not affect the primary outcome.

### Systematic review and meta-analysis of the association between common variants in *GCKR* and eGFR and CKD

Of the 661 eligible records that were selected by our initial search, eight studies fulfilled all in- and exclusion criteria and were used for the meta-analyses (see S2 Fig for flowchart and reasons for exclusion, and S5 Table for duplicate studies). All included studies were written in English. The genetic variants of interest were often not reported in the main article, but could be found in the (online) supplementary materials of the article. For two GWAS, the authors were contacted and the requested data were kindly provided [36, 37]. Six studies reported data on creatinine-based eGFR [36, 38–42], one on cystatin C-based eGFR [15], and four on CKD [36, 37, 40, 42]. Study characteristics of the eGFR and CKD studies are provided in Table 2. All studies used only the (creatinine-based) eGFR criterion to define CKD. Quality assessment of the included studies yielded an average score of five out of nine stars (S6 Table). Many studies reporting on eGFR scored low on ‘comparability’, i.e. the analyses were adjusted for covariates more than age and/or sex, whereas we aimed to obtain the crude relationship between *GCKR* and eGFR.

**Table 2:** Characteristics of included studies on eGFR and CKD.

	Author	Year	Ancestry	Population type	Number of cases*	Number of controls	Adjusted covariates	SNP	EAF	Definition of outcome
**eGFR** **(creatinine-based)**	**Bonetti [38]**	2011	European	T2D	474	-	Age, sex, BMI	rs780094	0.47	MDRD
	**Deshmukh [39]**	2013	European	T2D	2,970	-	Age, sex, BMI, SBP, HbA1c, T2DM duration	rs1260326		MDRD
	**Hishida [40]**	2014	Asian	General	3,324	-	Age, sex	rs1260326	0.61	Modified MDRD
	**Okada [41]**	2012	Asian	General + hospital	42,451	-	Age, sex, alcohol, smoking, BMI	rs1260326	0.52	Modified CKD-EPI
	**Pattaro [42]**	2016	European	General + T2D	133,413	-	Age, sex	rs1260326	0.42	MDRD
	**Yamada [36]**	2013	Asian	Hospital	12,563	-	Age, sex	rs1260326	0.57	Modified MDRD
**eGFR (cystatin C-based)**	**Köttgen [15]**	2010	European	General + T2D	20,907	-	Age, sex	rs1260326	0.41	76.7 × (serum cystatin c)−1.19
**CKD**	**Hishida [40]**	2014	Asian	General	578	2,746	-	rs1260326	0.61	eGFR < 60 ml/min/1.73m2
	**Pattaro [42]**	2016	European	General + T2D	12,385	104,780	Age, sex	rs1260326	0.42	eGFR < 60 ml/min/1.73m2
	**Svein-** **Bjornsson [37]**	2014	European	Hospital	15,594	291,428	Age, sex	rs1260326	0.35	eGFR < 60 ml/min/1.73m2
	**Yamada [36]**	2013	Asian	Hospital	3,085	9,478	Age, sex	rs1260326	0.57	eGFR < 50 ml/min/1.73m2

*Number of cases for the eGFR trait refers to the overall population.

Abbreviations: SNP: single nucleotide polymorphism; EAF: effect allele frequency; BMI: body mass index; SBP: systolic blood pressure; MDRD: modification of diet in renal disease; CKD-EPI: chronic kidney disease epidemiology collaboration.

**Fig 2:**
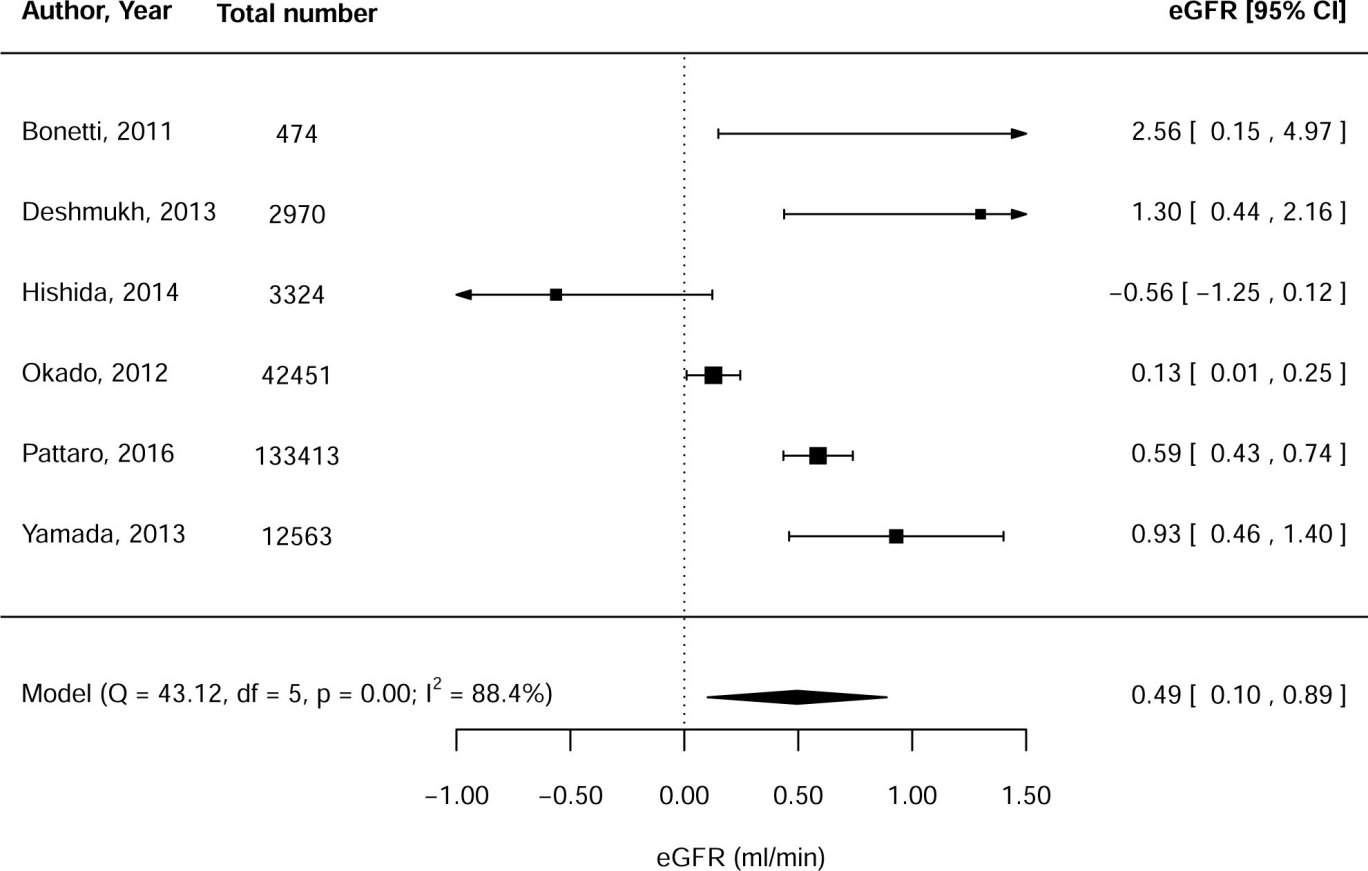
Meta-analysis of the relationship between the *GCKR* effect allele and creatinine-based estimated glomerular filtration rate (eGFR).

Meta-analysis, including 195,195 individuals, showed that the *GCKR* effect allele was significantly associated with an increased eGFR (0.49 ml/min, 95%CI: 0.10–0.89, p = 0.01) (Fig 2). Heterogeneity was high (Q = 43.12, I^2^ = 88.4%). The only study that reported on cystatin C-based eGFR observed similar effect sizes, which was statistically significant in the discovery cohort (p = 0.006), but not in the replication cohort (p = 0.16) [15].

**Fig 3:**
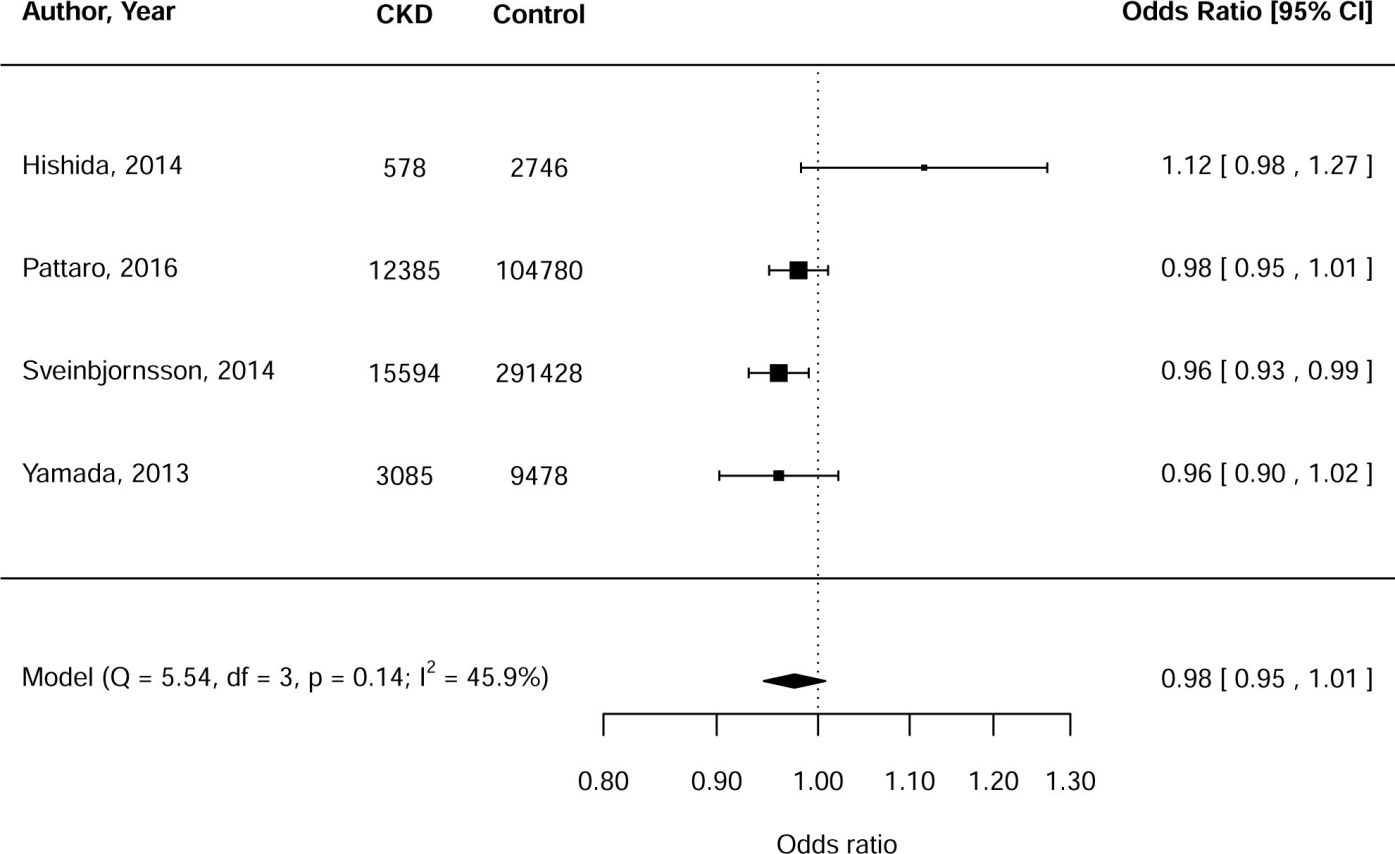
Meta-analysis of the relationship between the *GCKR* effect allele and chronic kidney disease (CKD).

The meta-analysis for CKD, including 31,642 cases and 408,432 controls, showed a protective effect of the *GCKR* effect allele on CKD, albeit not statistically significant (OR: 0.98, 95%CI: 0.95–1.01, p = 0.13; Q = 5.54, I^2^ = 45.9%) (Fig 3). The forest plot identified one outlying study that explained the moderate heterogeneity (Fig 3). Repeat analysis without this study [40] resulted in a significant, negative relationship (OR: 0.97, 95%CI: 0.95–0.99, p = 0.003). The same study also accounted for the non-significant relationship with CKD when sensitivity analyses were conducted for Asian studies only (S5 Fig). All CKD studies were of sufficient quality (NOS score ≥ 5 stars) and did not adjust for co-variates other than age and/or sex.

## Discussion

Glucokinase regulatory protein (GKRP) is a liver-specific protein that plays an important role in the regulation of hepatic glucose uptake and, consequently, de novo lipogenesis, one of the principal pathways in the development of NAFLD [11]. By studying the systemic effects of common variants in *GCKR* it is possible to gain more insight into the interaction between hepatic glucose metabolism and cardiorenal disease. Moreover, it allows an evaluation of small-molecule disruptors of the glucokinase-GKRP complex as a potential new glucose-lowering treatment. In three meta-analyses using data from at least ~200,000 individuals, we showed that the *GCKR* effect allele–which encodes a GKRP protein that binds glucokinase less effectively–appeared to be associated with CAD, whereas a protective effect was observed for eGFR.

Previous studies have shown that the *GCKR* effect allele is associated with an atherogenic lipid profile, i.e. higher plasma triglycerides and apolipoprotein B levels, reduced HDL cholesterol levels and the presence of small-dense LDL particles [12, 43, 44]. In that respect it is of no surprise that we did observe a positive association of *GCKR* on CAD in our primary analysis. If, however, one would take into account the effect of *GCKR* on only plasma triglycerides, it would be anticipated to already result in an odds ratio of 1.05 to develop CAD [45]. The smaller effect estimate that was found in this study (OR: 1.02, 95%CI: 1.00–1.04) should therefore be accounted for by another, protective factor that blunts the plasma lipid-mediated effects of *GCKR* on CAD risk. *GCKR* has previously been associated with reduced fasting plasma glucose levels [12]. The hitherto reported protective effect of *GCKR* on eGFR could be another explanatory factor. Previous epidemiological studies have shown that CKD is an independent cardiovascular risk factor [46].

The current meta-analyses were confined to creatinine-based renal outcome measures (eGFR and CKD), since these were most frequently reported. Köttgen and colleagues showed that the positive relationship between *GCKR* and (creatinine-based) eGFR was also observed for cystatin C-based eGFR [15]. The same authors suggested that another gene, which is in linkage disequilibrium with *GCKR*, is actually responsible for the association with renal function [15]. However, previous experiments in liver-specific glucokinase knockout mice–which are metabolically opposite to increased glucokinase-GKRP disruption–are characterized by *increased* kidney damage [47], which is in line with the current study.

The mechanism by which enhanced glucokinase-GKRP disruption exerts its renoprotective effects remains to be elucidated. The *GCKR* effect allele has been associated with increased NAFLD risk, low HDL cholesterol levels, and higher urate levels [12, 13, 43, 44, 48], which in turn have been associated with deterioration of renal function [49–51]. These factors should therefore be outbalanced by factors that protect the kidney, such as lower plasma glucose levels. We cannot exclude that there are also other, yet unknown factors that contribute to the renoprotective effect of the *GCKR* effect allele. Further research is needed to identify these factors as it may have important clinical implications.

The present study may provide a glimpse into the future of what the cardiorenal effects of small-molecule disruptors of the glucokinase-GKRP complex will be as a potential new glucose-lowering drug. Although the protective effect on eGFR and CKD appears to be promising at first sight, it may be outbalanced by an increased risk to develop CAD. Furthermore, a synergistic effect between *GCKR* and type 2 diabetes on CAD risk cannot be ruled out. We previously demonstrated that the effects of the *GCKR* effect allele on plasma lipid levels were more pronounced in patients with type 2 diabetes when compared to healthy individuals [52]. A similar interaction between *GCKR* and type 2 diabetes on CAD risk would seriously decrease the applicability of small molecule disruptors of the glucokinase-GKRP complex as new antidiabetic drug. Unfortunately, there were too few studies that were specifically conducted in type 2 diabetes to formally investigate such an interaction in the current meta-analysis.

This study has several strengths and limitations. First, the meta-analysis of the association of *GCKR* with CAD depends to a large extent on the the combined UK Biobank, CARDIoGRAMplusC4D 1000 genomes-based GWAS, and Myocardial Infarction Genetics and CARDIoGRAM Exome dataset, which is actually a meta-analysis by itself [31]. In subsequent sensitivity analyses we replaced this large dataset by other CARIoGRAMplusC4D-based studies that–despite a substantial overlap with the original study–included a large number of independent samples [32–34]. Although similar effect sizes were observed, statistical significance was not reached. The positive association between the *GCKR* effect allele and CAD in the primary analysis should therefore be interpreted with some caution.

Second, the definition of CKD was only based on eGFR–not the presence of albuminuria–in all of the included studies. Both factors are part of the classification of CKD as defined by the Kidney Disease Improving Global Outcomes (KDIGO) [53]. The CKD Genetics Consortium recently reported that the *GCKR* variant that protects from deterioration of renal function is associated with an *increased* urine albumin-creatinine ratio [51]. These findings emphasize the need for further research on the pathophysiological mechanisms relating GKRP to the kidney.

Third, it is not entirely clear whether the effects of genetic variants in *GCKR* and small molecule disruptors of the glucokinase-GKRP complex are truly comparable. This is one of the general limitations of the Mendelian randomization approach in which genetic variants are used as an instrument to study the effects of a specific drug of interest. However, previous experimental studies have shown that both the product of the *GCKR* minor allele and glucokinase-GKRP disruptors cause an increased translocation of glucokinase from the nucleus towards the cytosolic space in the liver [9, 17].This explains the reduced plasma glucose levels that have been associated with both the *GCKR* minor allele and treatment with glucokinase-GKRP disruptors [9, 54].

Another aspect that deserves consideration is the moderate-to-high heterogeneity that was observed in some of the meta-analyses. This could be the result of genotyping errors or difference in methodology, such as discrepancies in outcome measures (particularly for CAD) or study populations (e.g. population-based versus hospital-based). Although ancestry did not account for the moderate-to-high heterogeneity, the number of studies was too small to make strong inferences. Furthermore, differences in diet could contribute to the observed heterogeneity given the previously reported *GCKR*-diet interaction on plasma triglycerides levels [55, 56]. It is, however, unlikely that these factors truly account for the opposing effect sizes that were present in the individual studies, e.g. *GCKR* seemed to protect from CKD in one Japanese cohort [36, 41] whereas a predisposing effect appeared to be present in one other [40]. These opposing effects could simply be the consequence of chance, especially in small-sized studies with few events. Alternatively, *GCKR* could theoretically be in linkage disequilibrium with a gene that exerts an opposing effect on cardiorenal risk in certain but not all populations. These opposing effects could have important therapeutic implications if they would be inherent to GKRP function and therefore deserve further attention.

A final limitation was that we were forced to exclude a considerable amount of studies, and hence a substantial number of subjects, from the meta-analyses because of partial overlap of individual study cohorts. Yet, we were still able to include a high number of individuals, ranging from ~200,000 to 400,000 in the three meta-analyses, which can be attributed to our search strategy that was not confined to studies specifically reporting on *GCKR*. We correctly assumed that GWAS were likely to include our variants of interest without reporting in the manuscript’s title or abstract.

## Conclusions

The present study extends our knowledge on the systemic effects of enhanced disruption of the glucokinase-GKRP complex by demonstrating that the *GCKR* effect allele is associated with a better eGFR. A disadvantageous effect on CAD risk can, however, not be ruled out. These findings question the benefits and applicability of small molecule disruptors of the glucokinase-GKRP complex as a potential new class of antidiabetic drugs. Further studies are warranted toidentify the factor that mediates the renoprotective effects of enhanced disruption of the glucokinase-GKRP complex.

## Supporting information

S1 Table Search strategy for CAD. (DOCX)

S2 Table Search strategy for eGFR and CKD. (DOCX)

S3 Table Overview of the excluded CAD studies with duplicate cohorts. (DOCX)

S4 Table Quality assessment of the CAD studies based on the Newcastle-Ottawa Scale. (DOCX)

S5 Table Overview of the excluded eGFR and CKD studies with duplicate cohorts. (DOCX)

S6 Table Quality assessment of the eGFR and CKD studies based on the Newcastle-Ottawa Scale. (DOCX)

S1 Fig Flowchart of the systematic review on CAD. (DOCX)

S2 Fig Flowchart of the systematic review on eGFR and CKD. (DOCX)

S3 Fig Forest plot of the meta-analysis on CAD–sensitivity analysis. (DOCX)

S4 Fig Forest plot of the meta-analysis on CAD–stratified by ancestry. (DOCX)

S5 Fig Forest plot of the meta-analysis on CKD–stratified by ancestry. (DOCX)

S1 Checklist (DOCX)
