# Supplementary material for: Association of common gene variants in glucokinase regulatory protein with cardiorenal disease: A systematic review and meta-analysis
Source: PLoS One. 2018 Oct 23;13(10):e0206174. doi: 10.1371/journal.pone.0206174 (PMC6198948; doi:10.1371/journal.pone.0206174)
Supplement: S1 Table — (DOCX) [file pone.0206174.s001.docx]

**S1 Table. Search strategy for CAD**

Search strategy: MEDLINE (OVID) 1946 to 2018 week 10, EMBASE (OVID) 1974 to 2018 week 10.

Search strategy 1: CAD and *GCKR*

1. Acute coronary syndrome/
2. Coronary artery disease/
3. Ischemic heart disease/
4. Heart disease/
5. Coronary artery atherosclerosis/
6. Coronary artery occlusion/
7. Coronary artery thrombosis/
8. Cardiovascular disease/
9. Myocardial infarction/
10. Stroke/
11. Cerebrovascular accident/
12. Peripheral vascular disease/
13. Transient ischemic attack/
14. Peripheral arterial disease/
15. Acute coronary syndrome.mp
16. Coronary artery disease.mp
17. Ischemic heart disease.mp
18. Heart disease.mp
19. Coronary artery atherosclerosis.mp
20. Coronary artery occlusion.mp
21. Coronary artery thrombosis.mp
22. CAD.mp
23. Cardiovascular disease.mp
24. CVD.mp
25. Cardiac attack.mp
26. Heart attack.mp
27. Myocardial infarction.mp
28. MI.mp
29. Stroke.mp
30. Cerebrovascular accident.mp
31. Ischemic stroke.mp
32. Peripheral vascular disease.mp
33. Transient ischemic attack.mp
34. TIA.mp
35. Peripheral artery disease.mp
36. CVA.mp
37. GCKR.mp
38. GKRP.mp
39. Glucokinase regulatory protein.mp
40. Rs1260326.mp
41. P446L.mp
42. Rs780094.mp
43. Rs780093.mp
44. 1 or 2 or 3 or 4 or 5 or 6 or 7 or 8 or 9 or 10 or 11 or 12 or 13 or 14 or 15 or 16 or 17 or 18 or 19 or 20 or 21 or 22 or 23 or 24 or 25 or 26 or 27 or 28 or 29 or 30 or 31 or 32 or 33 or 34 or 35 or 36
45. 37 or 38 or 39 or 40 or 41 or 42 or 43
46. 44 and 45

*MEDLINE = 82 retrieved, EMBASE = 113 retrieved*

Search strategy 2: CAD and GWAS

1. Acute coronary syndrome/
2. Coronary artery disease/
3. Ischemic heart disease/
4. Heart disease/
5. Coronary artery atherosclerosis/
6. Coronary artery occlusion/
7. Coronary artery thrombosis/
8. Cardiovascular disease/
9. Myocardial infarction/
10. Stroke/
11. Cerebrovascular accident/
12. Peripheral vascular disease/
13. Transient ischemic attack/
14. Peripheral arterial disease/
15. Acute coronary syndrome.mp
16. Coronary artery disease.mp
17. Ischemic heart disease.mp
18. Heart disease.mp
19. Coronary artery atherosclerosis.mp
20. Coronary artery occlusion.mp
21. Coronary artery thrombosis.mp
22. CAD.mp
23. Cardiovascular disease.mp
24. CVD.mp
25. Cardiac attack.mp
26. Heart attack.mp
27. Myocardial infarction.mp
28. MI.mp
29. Stroke.mp
30. Cerebrovascular accident.mp
31. Ischemic stroke.mp
32. Peripheral vascular disease.mp
33. Transient ischemic attack.mp
34. TIA.mp
35. Peripheral artery disease.mp
36. CVA.mp
37. GWAS.mp
38. GWA study.mp
39. Genome-wide association study/
40. Genome wide association study.mp
41. Whole genome association study.mp
42. WGA study.mp
43. WGAS.mp
44. 1 or 2 or 3 or 4 or 5 or 6 or 7 or 8 or 9 or 10 or 11 or 12 or 13 or 14 or 15 or 16 or 17 or 18 or 19 or 20 or 21 or 22 or 23 or 24 or 25 or 26 or 27 or 28 or 29 or 30 or 31 or 32 or 33 or 34 or 35 or 36
45. 37 or 38 or 39 or 40 or 41 or 42 or 43
46. 43 and 45

*MEDLINE = 2026 retrieved, EMBASE = 1978 retrieved*
