# Supplementary material for: Association of common gene variants in glucokinase regulatory protein with cardiorenal disease: A systematic review and meta-analysis
Source: PLoS One. 2018 Oct 23;13(10):e0206174. doi: 10.1371/journal.pone.0206174 (PMC6198948; doi:10.1371/journal.pone.0206174)
Supplement: S2 Table — (DOCX) [file pone.0206174.s002.docx]

**S2 Table. Search strategy for eGFR and CKD**

Search strategy: MEDLINE (OVID) 1946 to 2018 week 10, EMBASE (OVID) 1974 to 2018 week 10.

Search strategy 1: eGFR, CKD and *GCKR*

1. Kidney disease/
2. Renal insufficiency, chronic/
3. Kidney failure, chronic/
4. Glomerular filtration rate/
5. Creatine/
6. Cystatin C/
7. Chronic kidney disease/
8. Glomerulopathy/
9. Kidney disease.mp
10. Chronic renal insufficiency.mp
11. Chronic kidney failure.mp
12. Glomerular filtration rate.mp
13. Creatine.mp
14. Cystatin C.mp
15. Chronic kidney disease.mp
16. CKD.mp
17. Renal disease.mp
18. Glomerulopathy.mp
19. GFR.mp
20. Estimated glomerular filtration rate.mp
21. eGFR.mp
22. GCKR.mp
23. GKRP.mp
24. Glucokinase regulatory protein.mp
25. Rs1260326.mp
26. P446L.mp
27. Rs780094.mp
28. Rs780093.mp
29. 1 or 2 or 3 or 4 or 5 or 6 or 7 or 8 or 9 or 10 or 11 or 12 or 13 or 14 or 15 or 16 or 17 or 18 or 19 or 20 or 21
30. 22 or 23 or 24 or 25 or 26 or 27 or 28
31. 29 and 30

*MEDLINE = 20 retrieved, EMBASE = 28 retrieved*

Search strategy 2: eGFR, CKD and GWAS

1. Kidney disease/
2. Renal insufficiency, chronic/
3. Kidney failure, chronic/
4. Glomerular filtration rate/
5. Creatine/
6. Cystatin C/
7. Chronic kidney disease/
8. Glomerulopathy/
9. Kidney disease.mp
10. Chronic renal insufficiency.mp
11. Chronic kidney failure.mp
12. Glomerular filtration rate.mp
13. Creatine.mp
14. Cystatin C.mp
15. Chronic kidney disease.mp
16. CKD.mp
17. Renal disease.mp
18. Glomerulopathy.mp
19. GFR.mp
20. Estimated glomerular filtration rate.mp
21. eGFR.mp
22. GWAS.mp
23. GWA study.mp
24. Genome-wide association study/
25. Genome wide association study.mp
26. Whole genome association study.mp
27. WGA study.mp
28. WGAS.mp
29. 1 or 2 or 3 or 4 or 5 or 6 or 7 or 8 or 9 or 10 or 11 or 12 or 13 or 14 or 15 or 16 or 17 or 18 or 19 or 20 or 21
30. 22 or 23 or 24 or 25 or 26 or 27 or 28
31. 29 and 30

*MEDLINE = 352 retrieved, EMBASE = 492 retrieved*
