# Supplementary material for: Association of common gene variants in glucokinase regulatory protein with cardiorenal disease: A systematic review and meta-analysis
Source: PLoS One. 2018 Oct 23;13(10):e0206174. doi: 10.1371/journal.pone.0206174 (PMC6198948; doi:10.1371/journal.pone.0206174)
Supplement: S3 Table — (DOCX) [file pone.0206174.s003.docx]

**S3 Table. Overview of the excluded CAD studies with duplicate cohorts**

| Excluded study | Study cohorts (with duplicate cohort in bold) | Study in which duplicate cohort is included |
| --- | --- | --- |
| Angelakopoulou (2012) [1] | Northwick Park Heart Study II (NPHS II), British Regional Heart Study (BRHS), English Longitudinal Study of Ageing (ELSA), Edinburgh Artery Study (EAS), Whitehall II study, The 1958 Birth Cohort (1958BC), The Medical Research Council National Survey of Health and Development (NSHD), Southampton Atherosclerosis Study (SAS), The Stockholm Heart Epidemiology Program (SHEEP), **The Wellcome Trust Case Control Consortium (WTCCC)**, University College Diabetes and Cardiovascular Study (UDACS), Ealing Diabetes Study of Coagulation (EDS), The MRC British Genetics of Hypertension (BRIGHT) | Nelson (2017) [2] |
| Bi (2010) [3] | **The atherosclerosis risk in communities (ARIC)*** | Nelson (2017) [2] |
| Burton (2007) [4] | **WTCCC**, 1958 British Birth Cohort, UK blood donor service | Nelson (2017) [2] |
| Davies (2012) [5] | **The Ottowa heart genomics study (OHGS)**, **Cleveland clinic gene bank (CCGB), WTCCC**, **INTERHEART**, **Duke Cathgen Study (DUKE)** | Nelson (2017) [2] |
| Dehghan (2016) [6] | **Age, gene/environment susceptibility-Reykjavik Study (AGES)***, **ARIC***, cardiovascular health study (CHS), **Family heart study (FHS)**, **Rotterdam study** | Nelson (2017) [2] |
| Deloukas (2013) [7] | **Artherosclerotic Disease, Vascular Function & Genetic Epidemiology study (ADVANCE)**, **The academic Medical Center Amsterdam Premature Atherosclerosis Study (AMC-PAS)**, Angio-Lueb/KORAF3, **Cardiogenics study (CARDIOGENICS)**, The Dietary, lifestyle and genetic determinants of obesity and metabolic syndrome study (DILGOM), **DUKE**, **Estonian genome center of university of Tartu (EGCUT GWAS, EGCUT metabochip)**, **The European prospective investigation into cancer (EPIC)**, **Functional genomic diagnostic tools for coronary artery disease (FGENTCARD**), Fragmin and Fast Revascularization (FRISCII-GLACIER), Gene x lifestyle interactions and compmlex traits involed in elevated disease risk (GLACIER), **The genetics of diabetes audit and research in Tayside Scotland (GoDARTS)**, **MRC/BHF heart protection study (HPS)**, **The INTERHEART study (ITH)**, **London life sciences population study (LOLIPOP)**, **Ludwigshafen Risk and cardiovascular health study and echonicoccus Multilocularis and internal diseases in Leutkirch study (LURIC-EMIL)**, METabolic Syndrome In Men (METSIM), **Monica, Risk, Genetics, Archiving and monograph (MORGAM-FIN, MORGAM-FRA, MORGAM-GER, MORAGM-ITA, MORGAM-UNK)**, **OHGS**, **Prospective Investigation of the Vasculature in Uppsala Seniors (PIVUS)**, Pfizer-MGH-Broad (PMD), **PopGEN**, **European collaborative study of the genetics of precocious coronary artery disease (PROCARDIS)**, **The Pakistan Risk of Myocardial Infarction Study (PROMIS GWAS, PROMIS Metabochip)**, SCARF-SHEEP, Swedish Twin Registry (STR), **The Hellenic study of interactions between Snps and eating in atherosclerosis susceptibility (THISEAS)**, **Uppsala longitudinal study of adult men (ULSAM), WTCCC CAD 2**, **COROGENE**, The Finnish cardiovascular study (FINCAVAS), **Genomics Research in Cardiovascular disease (GenRIC)** | Nelson (2017) [2] |
| Divers (2017) [8] | **African American Diabetes Heart Study (AA-DHS)**, Jackson Heart Study | Raffield (2015) [9] |
| Erdmann (2010) [10] | **German myocardial infarction family study (GerMIFS III)** | Nelson (2017) [2] |
| Howson (2017) [11] | **The Copenhagen Ischaemic Heart Disease Study (CIHDS)**, **The Copenhagen General Population Study (CGPS)**, **Copenhagen City Heart Study (CCHS),** **EPIC-CVD**, **Bangladesh Risk of Acute Vascular Events (BRAVE)**, **PROMIS**, **ARIC**, **Women’s Health Initiative (**WHI), **Myocardial infarction genetics consortium (MIGen)**, TAIwan metaboCHIp Consortium (TAICHI) | Nelson (2017) [2] |
| Kozian (2010) [12] | **LURIC** | Nelson (2017) [2] |
| Lu (2014) [13] | **Beijing Atherosclerosis Study (BAS)**, **China atherosclerosis study (CAS)**, CARDIOGRAM cohorts, China CHS, China Collaborative Study of Cardiovascular Epidemiology | Nelson (2017) [2] |
| Lettre (2011) [14] | **ARIC***, The coronary artery risk development in young adults (CARDIA), Cleveland family study (CFS), Jackson heart Study (JHS) and Multi-Ethnic Study of Atherosclerosis (MESA) | Nelson (2017) [2] |
| Nikpay (2015) [15] | **ADVANCE**, **AGES***, **ARIC***, **BAS (Beijing atherosclerosis study)**, **CARDIOGENICS**, **CAS**, **CCGB**, **COROGENE**, **DUKE, EGCUT**, **Family Heart Study (FamHS)***, **FGENTCARD**, **FHS**, **GenRIC**, **GerMIFS I-IV**, **GoDARTS**, **MRC/BHF Heart Protection Study (HPS)**, HSDSS, **BioMe Biobank Program**, **INTERHEART**, **LIFE-Heart**, **LOLIPOP**, **LURIC**, **case control study (MAYO-VDB)**, **Medstar cardiac catheterization study (MedStar)**, **MIGen**, **OHGS**, **Univeristy of Pennsylvania Medical Centre cardiac catherization study (PennCATH)**, **PIVUS**, **A subset of FINRISK cohort study (PREDICTCVD)**, **PROCARDIS**, **PROMIS**, **Prospective study of pravastatin in the elderly at risk (PROSPER)***, **Rotterdam Study***, **THISEAS**, **TWINGENE**, **ULSAM**, **Women’s genome health study (WGHS)**, **WTCCC** | Nelson (2017) [2] |
| O’Donnell (2011) [16] | **Cohorts for heart and aging research in genomic epidemiology (CHARGE**), genetic epidemiology network of arteriopathy study (GENOA) | Nelson (2017) [2] |
| Pfister (2011) [17] | **EPIC Norfolk** | Nelson (2017) [2] |
| Schunkert (2011) [18] | **ADVANCE**, **Coronary artery disease and omics (CADomics)**, **CHARGE**, Diabetes epidemiology: collaborative analysis of diagnostic criteria in Europe (deCODE CAD), **GerMIFS I**, **GerMIFS II**, **GerMIFS III**, **LURIC**/AtheroRemo 1, **LURIC**/AtheroRemo 2, **MedStar**, **MIGen**, **OHGS1**, **PennCATH**, **WTCCC**, Acute Myocardial Infarction Gene Study / Dortmund Health Study (AMI/DHS), **AMC-PAS**, Angio-Lueb/Gokard, CHAOS, **Cleveland Clinic GeneBank/OHGS2, EPIC-CAD**, GENDER, GraceGenetics, **INTERHEART**, Intermountain Heart Collaborative Study (IHCS), Irish Family Study (IFS), **Italian atherosclerosis, thrombosis and vascular biology study (IATVB)**, LEEDS, **Malmo Diet and cancer study-cardiovascular cohort (MDCS)**, **Mid**-America Mid-America Heart Insitute (MAHI), **PopGen**, SAS, Study of Myoarcial Infarction in Leiden (SMILE), SHEEP, The Emory Genebank Study, The Johns Hopkins GeneSTAR Research Program, The New Zealand CAD Study, **THISEAS**, UKMI, **Verona Heart Study (VHS)** | Nelson (2017) [2] |
| Stark (2009) [19] | **German MI Family Study** | Nelson (2017) [2] |
| The IBC 50K CAD Consortium (2011) [20] | **ARIC***, BLOODOMICS-Dutch (**AMC-PAS** + AGNES), BLOODOMICS-**German LURIC** + Mannheim), British Heart Foundation Family Heart Study **(BHF-FHS)**, The cardiovascular Health Study (CHS), CARDIA, **FHS**, **LOLIPOP**, MONICA-KORA, **PennCATH**, **PROCARDIS**, **PROMIS** | Nelson (2017) [2] |
| Trégouët (2009) [21] | **WTCCC** | Nelson (2017) [2] |
| Varbo (2011) [22] | **CCHS**, **CGPS**, **CIHDS** | Nelson (2017) [2] |
| Wang (2011) [23] | **GeneID population China** | Lian (2013) [24] |
| Webb (2017) [25] | **ATVB**, **BHF-FHS**, **Vanderbilt University Medical Center Biorepository (BioVU)**, **DUKE**, **EPIC**, **First-time incidence of myocardial infarction in the AC county 3 (FIA3)**, **GoDARTS**, **EGCUT**, **German CAD North**, **German CAD South, Nord-Trondelag health study (HUNT)**, **BioMe Biobank**, **MDC**, **Montreal heart institute study (MHI)**, **OHS**, **PAS-AMC**, **PennCath**, **PROCARDIS**, **VHS**, **WHI** | Nelson (2017) [2] |
| Wild (2012) [26] | The Gutenberg Heart Study (GHS), Atherogene Registry, Gutenberg Heart Express Study (GHSExpress), **CHARGE**, **GerMIFSI**, **GerMIFSII**, **MedStar**, **PennCATH**, **The MIGen consortium**, **WTCCC-CAD**, AngioLueb, Etude Cas-Témoin sur I’Infarctus du Myocarde (ECTIM), **LURIC**, **MORGAM**, **Popgen** | Nelson (2017) [2] |
| Willer (2008) [27] | **WTCCC** | Nelson (2017) [2] |
| Yaghootkar (2014) [28] | **ADVANCE**, CADomics, CHARGE, DeCode, **GERMifs**, **LURIC**, **MedStar**, **MIGen**, **OHGS**, **PennCATH**, **WTCCC** | Nelson (2017) [2] |
| Zhao (2017) [29] | **PROMIS**, Risk Assessment of cerebrovascular events study (RACE)**, BRAVE**, A prospective cohort to determine evnrionment and genetic determinants of metabolic syndrome related factors (EPIDREAM), **FINRISK**, **MedStar**, **MDC**, **PennCATH**, **LOLIPOP**, The Singapore Indian Eye Study (SINDI), **The Khatri Sikh Diabetes Study (SDS)**, TAICHI, BioBank Japan (BBJ) | Nelson (2017) [2] |

*Cohorts AGES, ARIC, FamHS, FHS, PROSPER, and Rotterdam Study are part of the CHARGE consortium

**References**

1. Angelakopoulou A, Shah T, Sofat R, Shah S, Berry DJ, Cooper J, et al. Comparative analysis of genome-wide association studies signals for lipids, diabetes, and coronary heart disease: Cardiovascular Biomarker Genetics Collaboration. European Heart Journal. 2012;33(3):393-407.

2. Nelson CP, Goel A, Butterworth AS, Kanoni S, Webb TR, Marouli E, et al. Association analyses based on false discovery rate implicate new loci for coronary artery disease. Nat Genet. 2017;49(9):1385-91. Epub 2017/07/18. doi: 10.1038/ng.3913. PubMed PMID: 28714975.

3. Bi M, Kao WH, Boerwinkle E, Hoogeveen RC, Rasmussen-Torvik LJ, Astor BC, et al. Association of rs780094 in GCKR with metabolic traits and incident diabetes and cardiovascular disease: the ARIC Study. PLoS One. 2010;5(7):e11690. Epub 2010/07/28. doi: 10.1371/journal.pone.0011690. PubMed PMID: 20661421.

4. The Wellcome Trust Case Control C. Genome-wide association study of 14,000 cases of seven common diseases and 3,000 shared controls. Nature. 2007;447:661. doi: 10.1038/nature05911

https://www.nature.com/articles/nature05911#supplementary-information.

5. Davies RW, Wells GA, Stewart AF, Erdmann J, Shah SH, Ferguson JF, et al. A genome-wide association study for coronary artery disease identifies a novel susceptibility locus in the major histocompatibility complex. Circulation Cardiovascular genetics. 2012;5(2):217-25. Epub 2012/02/10. doi: 10.1161/circgenetics.111.961243. PubMed PMID: 22319020; PubMed Central PMCID: PMCPMC3335297.

6. Dehghan A, Bis JC, White CC, Smith AV, Morrison AC, Cupples LA, et al. Genome-Wide Association Study for Incident Myocardial Infarction and Coronary Heart Disease in Prospective Cohort Studies: The CHARGE Consortium. PLOS ONE. 2016;11(3):e0144997. doi: 10.1371/journal.pone.0144997.

7. Deloukas P, Kanoni S, Willenborg C, Farrall M, Assimes TL, Thompson JR, et al. Large-scale association analysis identifies new risk loci for coronary artery disease. Nat Genet. 2013;45(1):25-33. Epub 2012/12/04. doi: 10.1038/ng.2480. PubMed PMID: 23202125; PubMed Central PMCID: PMCPMC3679547.

8. Divers J, Palmer ND, Langefeld CD, Brown WM, Lu L, Hicks PJ, et al. Genome-wide association study of coronary artery calcified atherosclerotic plaque in African Americans with type 2 diabetes. BMC Genetics. 2017;18 (1) (no pagination)(105).

9. Raffield LM, Cox AJ, Carr JJ, Freedman BI, Hicks PJ, Langefeld CD, et al. Analysis of a cardiovascular disease genetic risk score in the Diabetes Heart Study. Acta Diabetol. 2015;52(4):743-51. Epub 2015/02/24. doi: 10.1007/s00592-015-0720-5. PubMed PMID: 25700702; PubMed Central PMCID: PMCPMC4506855.

10. Erdmann J, Willenborg C, Nahrstaedt J, Preuss M, Konig IR, Baumert J, et al. Genome-wide association study identifies a new locus for coronary artery disease on chromosome 10p11.23. European heart journal. 2011;32(2):158-68. Epub 2010/11/23. doi: 10.1093/eurheartj/ehq405. PubMed PMID: 21088011.

11. Howson JMM, Zhao W, Barnes DR, Ho WK, Young R, Paul DS, et al. Fifteen new risk loci for coronary artery disease highlight arterial-wall-specific mechanisms. Nature Genetics. 2017;49(7):1113-9.

12. Kozian DH, Barthel A, Cousin E, Brunnhofer R, Anderka O, Marz W, et al. Glucokinase-activating GCKR polymorphisms increase plasma levels of triglycerides and free fatty acids, but do not elevate cardiovascular risk in the Ludwigshafen Risk and Cardiovascular Health Study. Hormone and metabolic research = Hormon- und Stoffwechselforschung = Hormones et metabolisme. 2010;42(7):502-6. doi: 10.1055/s-0030-1249637. PubMed PMID: 20352598.

13. Sevastianova K, Santos A, Kotronen A, Hakkarainen A, Makkonen J, Silander K, et al. Effect of short-term carbohydrate overfeeding and long-term weight loss on liver fat in overweight humans. Am J Clin Nutr. 2012;96(4):727-34. Epub 2012/09/07. doi: 10.3945/ajcn.112.038695. PubMed PMID: 22952180.

14. Lettre G, Palmer CD, Young T, Ejebe KG, Allayee H, Benjamin EJ, et al. Genome-Wide Association Study of Coronary Heart Disease and Its Risk Factors in 8,090 African Americans: The NHLBI CARe Project. PLOS Genetics. 2011;7(2):e1001300. doi: 10.1371/journal.pgen.1001300.

15. Nikpay M, Goel A, Won HH, Hall LM, Willenborg C, Kanoni S, et al. A comprehensive 1,000 Genomes-based genome-wide association meta-analysis of coronary artery disease. Nat Genet. 2015;47(10):1121-30. doi: 10.1038/ng.3396. PubMed PMID: 26343387; PubMed Central PMCID: PMC4589895.

16. Speliotes EK, Yerges-Armstrong LM, Wu J, Hernaez R, Kim LJ, Palmer CD, et al. Genome-wide association analysis identifies variants associated with nonalcoholic fatty liver disease that have distinct effects on metabolic traits. PLoS Genet. 2011;7(3):e1001324. Epub 2011/03/23. doi: 10.1371/journal.pgen.1001324. PubMed PMID: 21423719; PubMed Central PMCID: PMCPMC3053321.

17. Pfister R, Barnes D, Luben RN, Khaw KT, Wareham NJ, Langenberg C. Individual and cumulative effect of type 2 diabetes genetic susceptibility variants on risk of coronary heart disease. Diabetologia. 2011;54(9):2283-7. Epub 2011/06/04. doi: 10.1007/s00125-011-2206-5. PubMed PMID: 21638130.

18. Schunkert H, Konig IR, Kathiresan S, Reilly MP, Assimes TL, Holm H, et al. Large-scale association analysis identifies 13 new susceptibility loci for coronary artery disease. Nat Genet. 2011;43(4):333-8. Epub 2011/03/08. doi: 10.1038/ng.784. PubMed PMID: 21378990; PubMed Central PMCID: PMCPMC3119261.

19. Stark K, Reinhard W, Grassl M, Erdmann J, Schunkert H, Illig T, et al. Common Polymorphisms Influencing Serum Uric Acid Levels Contribute to Susceptibility to Gout, but Not to Coronary Artery Disease. PLoS ONE. 2009;4(11):e7729. doi: 10.1371/journal.pone.0007729. PubMed PMID: PMC2766838.

20. The IBCKCADC. Large-Scale Gene-Centric Analysis Identifies Novel Variants for Coronary Artery Disease. PLOS Genetics. 2011;7(9):e1002260. doi: 10.1371/journal.pgen.1002260.

21. Tregouet DA, Konig IR, Erdmann J, Munteanu A, Braund PS, Hall AS, et al. Genome-wide haplotype association study identifies the SLC22A3-LPAL2-LPA gene cluster as a risk locus for coronary artery disease. Nature genetics. 2009;41(3):283-5. Epub 2009/02/10. doi: 10.1038/ng.314. PubMed PMID: 19198611.

22. Varbo A, Benn M, Tybjaerg-Hansen A, Grande P, Nordestgaard BG. TRIB1 and GCKR polymorphisms, lipid levels, and risk of ischemic heart disease in the general population. Arteriosclerosis, Thrombosis, and Vascular Biology. 2011;31(2):451-7.

23. Wang F, Xu CQ, He Q, Cai JP, Li XC, Wang D, et al. Genome-wide association identifies a susceptibility locus for coronary artery disease in the Chinese Han population. Nature genetics. 2011;43(4):345-9. Epub 2011/03/08. doi: 10.1038/ng.783. PubMed PMID: 21378986.

24. Lian J, Guo J, Chen Z, Jiang Q, Ye H, Huang X, et al. Positive association between GCKR rs780093 polymorphism and coronary heart disease in the aged Han Chinese. Dis Markers. 2013;35(6):863-8. Epub 2014/01/05. doi: 10.1155/2013/215407. PubMed PMID: 24385677; PubMed Central PMCID: PMCPMC3871702.

25. Webb TR, Erdmann J, Stirrups KE, Stitziel NO, Masca NG, Jansen H, et al. Systematic Evaluation of Pleiotropy Identifies 6 Further Loci Associated With Coronary Artery Disease. Journal of the American College of Cardiology. 2017;69(7):823-36. Epub 2017/02/18. doi: 10.1016/j.jacc.2016.11.056. PubMed PMID: 28209224; PubMed Central PMCID: PMCPMC5314135.

26. Wild PS, Zeller T, Schillert A, Szymczak S, Sinning CR, Deiseroth A, et al. A genome-wide association study identifies LIPA as a susceptibility gene for coronary artery disease. Circulation Cardiovascular genetics. 2011;4(4):403-12. Epub 2011/05/25. doi: 10.1161/circgenetics.110.958728. PubMed PMID: 21606135; PubMed Central PMCID: PMCPMC3157552.

27. Willer CJ, Sanna S, Jackson AU, Scuteri A, Bonnycastle LL, Clarke R, et al. Newly identified loci that influence lipid concentrations and risk of coronary artery disease. Nat Genet. 2008;40(2):161-9. doi: 10.1038/ng.76. PubMed PMID: 18193043.

28. Yaghootkar H, Scott RA, White CC, Zhang W, Speliotes E, Munroe PB, et al. Genetic evidence for a normal-weight "metabolically obese" phenotype linking insulin resistance, hypertension, coronary artery disease, and type 2 diabetes. Diabetes. 2014;63(12):4369-77. Epub 2014/07/23. doi: 10.2337/db14-0318. PubMed PMID: 25048195; PubMed Central PMCID: PMCPMC4392920.

29. Zhao B, Lu Q, Cheng Y, Belcher JM, Siew ED, Leaf DE, et al. A Genome-wide association study to identify single-nucleotide polymorphisms for acute kidney injury. American Journal of Respiratory and Critical Care Medicine. 2017;195(4):482-90.
