# Supplementary material for: Association of common gene variants in glucokinase regulatory protein with cardiorenal disease: A systematic review and meta-analysis
Source: PLoS One. 2018 Oct 23;13(10):e0206174. doi: 10.1371/journal.pone.0206174 (PMC6198948; doi:10.1371/journal.pone.0206174)
Supplement: S4 Table — (DOCX) [file pone.0206174.s004.docx]

**S4 Table. Quality assessment of the CAD studies based on the Newcastle-Ottawa Scale**

| **References** | **Selection** | | | | **Comparability** | **Exposure/Outcome** | | | | **Quality judgment** |
| --- | --- | --- | --- | --- | --- | --- | --- | --- | --- | --- |
|  | **1** | **2** | **3** | **4** | **1** | | **1** | **2** | **3** |  |
| Lian (2013) [1] |  |  |  |  |  | |  |  |  |  |
| Nelson (2017) [2] |  |  |  |  |  | |  |  |  |  |
| Raffield (2015) [3] |  |  |  |  |  | |  |  |  |  |
| Takeuchi (2012) [4] |  |  |  |  |  | |  |  |  |  |
| Zhou (2015) [5] |  |  |  |  |  | |  |  |  |  |

Notes case-control studies (i.e. Lian (2013), Takeuchi (2012), Zhou (2015)): categories of the quality assessment are displayed in bold, with interpretation of each item within the categories for this specific meta-analysis placed between brackets.

**Selection**: 1. Is the case definition adequate? (if yes, with independent validation (e.g. hospital records), one star; if yes, with record linkage (e.g. ICD-10 code or self-report) or no description, no star); 2. Representativeness of the cases (if consecutive or obviously representative series of cases, one star; if not consecutive or not (clearly) stated, no star); 3. Selection of controls (if community controls, one star; if hospital controls or no description, no star); 4. Definition of controls (if yes, with ‘no history of CAD’ explicitly stated, one star; if ‘no history of CAD’ not explicitly stated or no description, no star). **Comparability**: 1. Comparability of cases and controls on the basis of the design or analysis (if study adjusts for no covariates, two stars, if study adjusts for age and/or gender only, one star, if study adjusts for more covariates than age and/or gender, no star). **Exposure**: 1. Ascertainment of exposure (if secure record (e.g. genotyping), one star; if no description, no star); 2. Same method of ascertainment for cases and controls (if yes, one star; if no or no description, no star); 3. Non-response rate (if same rate for both groups, one star; if rate differs for both groups or no designation, no star).

Notes cohort studies (i.e. Nelson (2017), Raffield (2015)): categories of the quality assessment are displayed in bold, with interpretation of each item within the categories for this specific meta-analysis placed between brackets.

**Selection**: 1. Representativeness of the exposed cohort (if truly or somewhat representative of the average population, one star; if selected group (e.g. patients with type 2 diabetes) or no description, no star). 2. Selection of the non-exposed cohort (if drawn from the same community as the exposed cohort, one star; if drawn from a different source or no description, no star). 3. Ascertainment of exposure (if secure record (e.g. genotyping), one star; if no description, no star). 4. Demonstration that outcome of interest was not present at the start of the study (if explicitly stated, one star; if not explicitly stated, no star). **Comparability**: 1. Comparability of cases and controls on the basis of the design or analysis (if study adjusts for no covariates, two stars, if study adjusts for age and/or gender only, one star, if study adjusts for more covariates than age and/or gender, no star). **Outcome**: 1. Assessment of outcome (if independent blind assessment or record linkage (e.g. hospital records), one star; if with record linkage (e.g. ICD-10 code or self-report) or no description, no star). 2. Was follow-up long enough for outcome to occur (if average age of the sample population minus two standard deviations was equal to or more than 40 years, one star; if average age of the sample population minus two standard deviations was less than 40 years, no star). 3. Adequacy of follow up of cohorts (if explicitly stated why subjects from original cohort were excluded, one star; if not explicitly stated why subjects from original cohort were excluded, no star).

**References**

1. Lian J, Guo J, Chen Z, Jiang Q, Ye H, Huang X, et al. Positive association between GCKR rs780093 polymorphism and coronary heart disease in the aged Han Chinese. Dis Markers. 2013;35(6):863-8. Epub 2014/01/05. doi: 10.1155/2013/215407. PubMed PMID: 24385677; PubMed Central PMCID: PMCPMC3871702.

2. Nelson CP, Goel A, Butterworth AS, Kanoni S, Webb TR, Marouli E, et al. Association analyses based on false discovery rate implicate new loci for coronary artery disease. Nat Genet. 2017;49(9):1385-91. Epub 2017/07/18. doi: 10.1038/ng.3913. PubMed PMID: 28714975.

3. Raffield LM, Cox AJ, Carr JJ, Freedman BI, Hicks PJ, Langefeld CD, et al. Analysis of a cardiovascular disease genetic risk score in the Diabetes Heart Study. Acta Diabetol. 2015;52(4):743-51. Epub 2015/02/24. doi: 10.1007/s00592-015-0720-5. PubMed PMID: 25700702; PubMed Central PMCID: PMCPMC4506855.

4. Takeuchi F, Isono M, Katsuya T, Yokota M, Yamamoto K, Nabika T, et al. Association of genetic variants influencing lipid levels with coronary artery disease in Japanese individuals. PLoS One. 2012;7(9):e46385. Epub 2012/10/11. doi: 10.1371/journal.pone.0046385. PubMed PMID: 23050023; PubMed Central PMCID: PMCPMC3458872.

5. Zhou YJ, Hong SC, Yin RX, Yang Q, Cao XL, Chen WX. Polymorphisms in the GCKR are associated with serum lipid traits, the risk of coronary artery disease and ischemic stroke. Int J Clin Exp Med. 2015;8(7):10678-86. Epub 2015/09/18. PubMed PMID: 26379859; PubMed Central PMCID: PMCPMC4565242.
