# Supplementary material for: Association of common gene variants in glucokinase regulatory protein with cardiorenal disease: A systematic review and meta-analysis
Source: PLoS One. 2018 Oct 23;13(10):e0206174. doi: 10.1371/journal.pone.0206174 (PMC6198948; doi:10.1371/journal.pone.0206174)
Supplement: S5 Table — (DOCX) [file pone.0206174.s005.docx]

**S5 Table. Overview of the excluded eGFR and CKD studies with duplicate cohorts**

| Excluded study | Study cohorts (with duplicate cohort in bold) | Study in which duplicate cohort is included |
| --- | --- | --- |
| Gorski (2015) [1] | **Age, Gene/Environment susceptibility-Reykjavik Study (AGES)***, **Amish Studies**, **The atherosclerosis risk in communities study (ARIC)***, **Austrian stroke prevention study (ASPS)**, **Cardiovascular health study (CHS)**, The Cohort Lausannoise study (CoLaus), **Framingham Heart Study (FHS)**, **Genetic epidemiology network of arteriopathy (GENOA)**, **Health aging and body composition study (HABC)**, **JUPITER**, **Cooperative research in the region of Augsburg (KORA)**, **multi-ethnic study of atherosclerosis (MESA)**, **Rotterdam Study**, **Study of health in Pomerania (SHIP)**, **Three Cities (3C)** | Pattaro (2016) [2] |
| Köttgen (2009) [3] | Cohorts for heart and aging research in genomic epidemiology (CHARGE) which includes **ARIC***, **CHS**, **FHS**, **Rotterdam Study** | Pattaro (2016) [2] |
| Köttgen (2010) [4]** | **AGES***, **Amish studies**, **ARIC***, **ASPS**, **Baltimore longitudinal study of aging (BLSA)**, **CHS**, **Erasmus rucphen family (ERF)**, **FamHS***, **FHS**, **GENOA**, Gutenburg Heart Study (GHS), **KORA F3 and F4**, **Korcula Croatia**, **Microisolates in South tyrol study (MICROS)**, **The northern Swedish population health study (NSPHS)**, **Orkney complex disease study (ORCADES)**, **Rotterdam Study**, **SHIP**, VIS **CROATIA**, **Women’s genome health study (WGHS)**, **HABC**, **Health professionals follow-up study (HPFS)**, **Nurses health study (NHS)**, **POPGen**, **The Sorbs study (Sorbs)**, SPLIT, **Swiss study on air polluation and lung diseases in adults (SAPALDIA)**, **Salzburg Atherosclerosis prevention program in subjects at high individual risk (SAPHIR)** | Pattaro (2016) [2] |
| Lanktree (2018) [5] | **Atherosclerotic Disease, Vascular function, & Genetic epidemiology study (ADVANCE)**, The academic medical center of Amsterdam Premature Atherosclerosis Cohort (AMC-PAS), **AMISH**, 1958 British Birth Cohort (BC58), D2D 2007 (D2D), deCODE, The diabetes Genetic Study (DIAGEN), The dietary, lifestyle and genetic determinants of obesity and metabolic syndrome study (DILGOM), The finnish diabetes prevention study (DPS), The dose responses to exercise training study (DR’S EXTRA), Edinburgh Artery Study (EAS), **Estonian genome center of university of Tartu (EGCUT)**, Ely, The european prospective investigation into cancer and nutrition (EPIC-CAD), Fenland, The Finnish cardiovascular study (FINCAVAS), Fragmin and fast revascularization during instability in coronary artery disease (FRISCII), FUSION2, Gene x lifestyle interactions and complex traits involved in elevated disease risk (GLACIER), **Genetics of diabetes and audit research Tayside (Go-DARTs)**, Nord-Trondelag health study 2 (HUNT), IMPROVE, **KORA F3 AND F4**, Ludwigshafen Risk and Cardiovascular Health Study (LURIC), Malmo diet and cancer study (MDC), Metabolic syndrome in men (METSIM), Northern Finland birth cohort 1986 (NFBC1986), MRC national survey of health and development (NSHD), Prospective investigation of the vasculature in Uppsale seniors (PIVUS), Sardinia study on aging (SardiNIA), SCARFSHEEP, Swedish Twin Register (STR), The Hellenic study of interactions between SNPs and eating in atherosclerosis susceptibility (THISEAS), TROMSO, Uppsala longitudinal study of adult men (ULSAM), Whitehall II, Cebu longitudinal health and nutrition survey (CLHNS), Taiwan metabochip consortium (TAICHI), Asian Indian diabetic heart study/Sikh diabetes study (AIDHS/SDS), The Pakistan risk of myocardial infarction study (PROMIS), Family blood pressure project GenNet and HyperGen studies (FBPP), Kingston GXE (GXE), General population cohort study, Uganda (MRC/UVRI GPC), Seychelles tandem study (SEY), Spanishtown (SPT), **AGES**, **ARIC**, TWIN COHORTS, **BLSA**, **CHS**, CoLaus, Invecchiare in Chianti study (InCHIANTI), London life sciences prospective population study (LOLIPOP), National FINRISK Study, PARC, **Rotterdam Study**, Supplementation en vitamins et mineraux antioxydants study (SUMIVAX), **WGHS**, British genetics of hypertension study (BRIGHT), Britisch 1958 birth cohort type 1 diabetes genetics consortium (B58CT1DGC), Diabetes genetics initiative (DGI), FHS, HEATLH2000 GenMets Study, MedStar, PennCATH, **ERF**, Framingham Heart Study (FramHS), **MICROS**, **NSPHS**, **ORCADES**, Vis Study, National FINRISK 1997 study (FINRISK97), Coronary artery disease genomewide replication and meta-analysis study (CARDIoGRAM), COROGENE | Pattaro (2016) [2]  Deshmukh (2013) [6] |
| Li (2017) [7] | **AGES**, **Amish**, **ARIC**, **CHS**, **CROATIA-Korcula**, **EGCUT**, **FamHS**, **FHS**, Geisinger Genomic Medicine Exome project (GeMEP), Generation Scotland: Scottish family health study (GS:SFHS), **HPFS**, Health and retirement study (HRS), **European network for genetic-epidemiological studies (HYPERGENES)**, **Italian network on genetic isolates (INGI)**, Mount Sinai BioMe Biobank program (IPM), **KORA F4**, METSIM, **NHS**, **Rotterdam Study**, **SAPALDIA**, **SHIP**, **WGHS**, Women’s health initiative (WHI), **Young Finns Study (YFS)**, **ARIC**, **CHS**, **GENOA**, Jackson Heart Study (JHS) | Pattaro (2016) [2] |
| Pattaro (2012) [8] | **AGES***, **AMISCH**, SPS, **ARIC***, **BLSA**, **CHS**, **ERF**, **FamHS***, **FHS**, **GENOA**, **HABC**, **HPFS**, **KORA** **F3 and F4**, **Croatia-Korcula cohort**, **MICROS**, **NSPHS**, **NHS**, **ORCADES**, **POPGen**, **Sorbs**, **Rotterdam study**, **SHIP**, VIS, **WGHS** | Pattaro (2016) [2] |
| Thio (2017) [9] | **Prevention of renal and vascular endstage disease (PREVEND)** | Pattaro (2016)[ 2] |

*Cohorts AGES, ARIC, FamHS, FHS, PROSPER, and Rotterdam Study are part of the CHARGE consortium

**This study was included for the data-synthesis on cystatin C-based eGFR, since this was not reported by Pattaro (2016) [33]

**References**

1. Gorski M, Tin A, Garnaas M, McMahon GM, Chu AY, Tayo BO, et al. Genome-wide association study of kidney function decline in individuals of European descent. Kidney international. 2015;87(5):1017-29. Epub 2014/12/11. doi: 10.1038/ki.2014.361. PubMed PMID: 25493955; PubMed Central PMCID: PMCPMC4425568.

2. Pattaro C, Teumer A, Gorski M, Chu AY, Li M, Mijatovic V, et al. Genetic associations at 53 loci highlight cell types and biological pathways relevant for kidney function. Nat Commun. 2016;7:10023. Epub 2016/02/03. doi: 10.1038/ncomms10023. PubMed PMID: 26831199; PubMed Central PMCID: PMCPMC4735748.

3. Köttgen A, Glazer NL, Dehghan A, Hwang S-J, Katz R, Li M, et al. Multiple Novel Loci are Associated with Indices of Renal Function and Chronic Kidney Disease. Nature genetics. 2009;41(6):712-7. doi: 10.1038/ng.377. PubMed PMID: PMC3039280.

4. Kottgen A, Pattaro C, Boger CA, Fuchsberger C, Olden M, Glazer NL, et al. New loci associated with kidney function and chronic kidney disease. Nat Genet. 2010;42(5):376-84. Epub 2010/04/13. doi: 10.1038/ng.568. PubMed PMID: 20383146; PubMed Central PMCID: PMCPMC2997674.

5. Lanktree MB, Theriault S, Walsh M, Pare G. HDL Cholesterol, LDL Cholesterol, and Triglycerides as Risk Factors for CKD: A Mendelian Randomization Study. American journal of kidney diseases : the official journal of the National Kidney Foundation. 2018;71(2):166-72. Epub 2017/07/30. doi: 10.1053/j.ajkd.2017.06.011. PubMed PMID: 28754456.

6. Deshmukh HA, Palmer CN, Morris AD, Colhoun HM. Investigation of known estimated glomerular filtration rate loci in patients with type 2 diabetes. Diabet Med. 2013;30(10):1230-5. Epub 2013/04/17. doi: 10.1111/dme.12211. PubMed PMID: 23586973; PubMed Central PMCID: PMCPMC4204276.

7. Li M, Li Y, Weeks O, Mijatovic V, Teumer A, Huffman JE, et al. SOS2 and ACP1 Loci Identified through Large-Scale Exome Chip Analysis Regulate Kidney Development and Function. Journal of the American Society of Nephrology : JASN. 2017;28(3):981-94. Epub 2016/12/07. doi: 10.1681/asn.2016020131. PubMed PMID: 27920155; PubMed Central PMCID: PMCPMC5328154.

8. Pattaro C, Köttgen A, Teumer A, Garnaas M, Böger CA, Fuchsberger C, et al. Genome-Wide Association and Functional Follow-Up Reveals New Loci for Kidney Function. PLOS Genetics. 2012;8(3):e1002584. doi: 10.1371/journal.pgen.1002584.

9. Thio CHL, van der Most PJ, Nolte IM, van der Harst P, Bultmann U, Gansevoort RT, et al. Evaluation of a genetic risk score based on creatinine-estimated glomerular filtration rate and its association with kidney outcomes. Nephrology, dialysis, transplantation : official publication of the European Dialysis and Transplant Association - European Renal Association. 2017. Epub 2018/01/03. doi: 10.1093/ndt/gfx337. PubMed PMID: 29294079.
