# Supplementary material for: Association of common gene variants in glucokinase regulatory protein with cardiorenal disease: A systematic review and meta-analysis
Source: PLoS One. 2018 Oct 23;13(10):e0206174. doi: 10.1371/journal.pone.0206174 (PMC6198948; doi:10.1371/journal.pone.0206174)
Supplement: S6 Table — (DOCX) [file pone.0206174.s006.docx]

**S6 Table. Quality assessment of the eGFR and CKD studies based on the Newcastle-Ottawa Scale**

| **References** | **Selection** | | | | | **Comparability** | | **Exposure/ Outcome** | | | | **Quality judgment** | |
| --- | --- | --- | --- | --- | --- | --- | --- | --- | --- | --- | --- | --- | --- |
|  | **1** | **2** | **3** | **4** | **1** | | **1** | | **2** | **3** |  | |  |
| Bonetti (2011) [1] |  |  |  |  |  | |  | |  |  |  | |  |
| Deshmukh (2013) [2] |  |  |  |  |  | |  | |  |  |  | |  |
| Hishida (2014) [3] |  |  |  |  |  | |  | |  |  |  | |  |
| Köttgen (2010) [4] |  |  |  |  |  | |  | |  |  |  | |  |
| Okada (2012) [5] |  |  |  |  |  | |  | |  |  |  | |  |
| Pattaro (2016) [6] |  |  |  |  |  | |  | |  |  |  | |  |
| Sveinbjornsson (2014) [7] |  |  |  |  |  | |  | |  |  |  | |  |
| Yamada (2013) [8] |  |  |  |  |  | |  | |  |  |  | |  |

Notes case-control studies (i.e. Sveinbjornsson (2014), Yamada (2013)): categories of the quality assessment are displayed in bold, with interpretation of each item within the categories for this specific meta-analysis placed between brackets.

**Selection**: 1. Is the case definition adequate? (if yes, with independent validation (e.g. hospital records), one star; if yes, with record linkage (e.g. ICD-10 code or self-report) or no description, no star); 2. Representativeness of the cases (if consecutive or obviously representative series of cases, one star; if not consecutive or not (clearly) stated, no star); 3. Selection of controls (if community controls, one star; if hospital controls or no description, no star); 4. Definition of controls (if yes, with ‘no history of CKD’ explicitly stated, one star; if ‘no history of CKD’ not explicitly stated or no description, no star). **Comparability**: 1. Comparability of cases and controls on the basis of the design or analysis (if study adjusts for no covariates, two stars, if study adjusts for age and/or gender only, one star, if study adjusts for more covariates than age and/or gender, no star). **Exposure**: 1. Ascertainment of exposure (if secure record (e.g. genotyping), one star; if no description, no star); 2. Same method of ascertainment for cases and controls (if yes, one star; if no or no description, no star); 3. Non-response rate (if same rate for both groups, one star; if rate differs for both groups or no designation, no star).

Notes cohort studies (i.e. Bonetti (2011), Deshmukh (2013), Hishida (2014), Köttgen (2010), Okado (2012), Pattaro (2016)): categories of the quality assessment are displayed in bold, with interpretation of each item within the categories for this specific meta-analysis placed between brackets.

**Selection**: 1. Representativeness of the exposed cohort (if truly or somewhat representative of the average population, one star; if selected group (e.g. patients with type 2 diabetes) or no description, no star). 2. Selection of the non-exposed cohort (if drawn from the same community as the exposed cohort, one star; if drawn from a different source or no description, no star). 3. Ascertainment of exposure (if secure record (e.g. genotyping), one star; if no description, no star). 4. Demonstration that outcome of interest was not present at the start of the study (if dichotomous variable (e.g. CKD yes/no) and ‘no history of CKD’ explicitly stated, one star; if dichotomous variable (e.g. CKD yes/no) and ‘no history of CKD’ not explicitly stated, or continuous variable (e.g. eGFR), no star). **Comparability**: 1. Comparability of cases and controls on the basis of the design or analysis (if study adjusts for no covariates, two stars, if study adjusts for age and/or gender only, one star, if study adjusts for more covariates than age and/or gender, no star). **Outcome**: 1. Assessment of outcome (if independent blind assessment or record linkage (e.g. hospital records), one star; if with record linkage (e.g. ICD-10 code or self-report) or no description, no star). 2. Was follow-up long enough for outcome to occur (if average age of the sample population minus two standard deviations was equal to or more than 40 years or variable was continuous (e.g. eGFR), one star; if average age of the sample population minus two standard deviations was less than 40 years, no star). 3. Adequacy of follow up of cohorts (if explicitly stated why subjects from original cohort were excluded, one star; if not explicitly stated why subjects from original cohort were excluded, no star).

**References**

1. Bonetti S, Trombetta M, Boselli ML, Turrini F, Malerba G, Trabetti E, et al. Variants of GCKR affect both beta-cell and kidney function in patients with newly diagnosed type 2 diabetes: the Verona newly diagnosed type 2 diabetes study 2. Diabetes Care. 2011;34(5):1205-10. Epub 2011/03/18. doi: 10.2337/dc10-2218. PubMed PMID: 21411509; PubMed Central PMCID: PMCPMC3114499.

2. Deshmukh HA, Palmer CN, Morris AD, Colhoun HM. Investigation of known estimated glomerular filtration rate loci in patients with type 2 diabetes. Diabet Med. 2013;30(10):1230-5. Epub 2013/04/17. doi: 10.1111/dme.12211. PubMed PMID: 23586973; PubMed Central PMCID: PMCPMC4204276.

3. Hishida A, Takashima N, Turin TC, Kawai S, Wakai K, Hamajima N, et al. GCK, GCKR polymorphisms and risk of chronic kidney disease in Japanese individuals: data from the J-MICC Study. J Nephrol. 2014;27(2):143-9. Epub 2014/02/19. doi: 10.1007/s40620-013-0025-0. PubMed PMID: 24535998.

4. Kottgen A, Pattaro C, Boger CA, Fuchsberger C, Olden M, Glazer NL, et al. New loci associated with kidney function and chronic kidney disease. Nat Genet. 2010;42(5):376-84. Epub 2010/04/13. doi: 10.1038/ng.568. PubMed PMID: 20383146; PubMed Central PMCID: PMCPMC2997674.

5. Okada Y, Sim X, Go MJ, Wu JY, Gu D, Takeuchi F, et al. Meta-analysis identifies multiple loci associated with kidney function-related traits in east Asian populations. Nat Genet. 2012;44(8):904-9. Epub 2012/07/17. doi: 10.1038/ng.2352. PubMed PMID: 22797727; PubMed Central PMCID: PMCPMC4737645.

6. Pattaro C, Teumer A, Gorski M, Chu AY, Li M, Mijatovic V, et al. Genetic associations at 53 loci highlight cell types and biological pathways relevant for kidney function. Nat Commun. 2016;7:10023. Epub 2016/02/03. doi: 10.1038/ncomms10023. PubMed PMID: 26831199; PubMed Central PMCID: PMCPMC4735748.

7. Sveinbjornsson G, Mikaelsdottir E, Palsson R, Indridason OS, Holm H, Jonasdottir A, et al. Rare mutations associating with serum creatinine and chronic kidney disease. Hum Mol Genet. 2014;23(25):6935-43. Epub 2014/08/02. doi: 10.1093/hmg/ddu399. PubMed PMID: 25082825.

8. Yamada Y, Nishida T, Ichihara S, Kato K, Fujimaki T, Oguri M, et al. Identification of chromosome 3q28 and ALPK1 as susceptibility loci for chronic kidney disease in Japanese individuals by a genome-wide association study. J Med Genet. 2013;50(6):410-8. Epub 2013/03/30. doi: 10.1136/jmedgenet-2013-101518. PubMed PMID: 23539754.
