# Supplementary material for: Association of common gene variants in glucokinase regulatory protein with cardiorenal disease: A systematic review and meta-analysis
Source: PLoS One. 2018 Oct 23;13(10):e0206174. doi: 10.1371/journal.pone.0206174 (PMC6198948; doi:10.1371/journal.pone.0206174)
Supplement: S1 Fig — (DOCX) [file pone.0206174.s007.docx]

**S1 Fig. Flowchart of the systematic review on CAD**

Records identified through database searching (1)

n=195

Records identified through database searching (2)

n=4004

Records after duplicates removed

n=3051

Records screened on title and/or abstract

n=3051

Records excluded on title and/or abstract, reasons: language, topic, no original article

n=2936

Full-text records assessed for eligibility

n=115

Records excluded (n=104), reasons:

- No original article (n=5)
- Duplicate cohort (n=26)
- *GCKR* not assessed (n=1)
- Other outcome measure (n=5)
- Family-based study (n=1)
- Other language (n=1)
- No GWAS (n=63)
- Longitudinal study (n=2)

Studies included in qualitative synthesis

n=11

Studies included in quantitative synthesis (i.e. meta-analysis)

n=5

Corresponding authors of GWAS contacted for information on *GCKR* (n=6), no reply:

n=6

Identification

Screening

Eligibility

Included
