# Supplementary material for: Association of common gene variants in glucokinase regulatory protein with cardiorenal disease: A systematic review and meta-analysis
Source: PLoS One. 2018 Oct 23;13(10):e0206174. doi: 10.1371/journal.pone.0206174 (PMC6198948; doi:10.1371/journal.pone.0206174)
Supplement: S2 Fig — (DOCX) [file pone.0206174.s008.docx]

**S2 Fig. Flowchart of the systematic review on eGFR and CKD**

Records identified through database searching (1)

n=48

Records identified through database searching (2)

n=844

Records after duplicates removed

n=661

Records screened on title and/or abstract

n=661

Records excluded on title and/or abstract, reasons: language, topic, no original article

n=609

Full-text records assessed for eligibility

n=52

Records excluded (n=43), reasons:

- No original article (n=14)
- Full-text not available (n=1)
- Duplicate cohort (n=7)
- *GCKR* not assessed (n=16)
- Other outcome measure (n=4)
- Family-based study (n=1)

Studies included in qualitative synthesis

n=9

Studies included in quantitative synthesis (i.e. meta-analysis)

n=8

Corresponding authors of GWAS contacted for information on *GCKR* (n=2), no reply:

n=1

Identification

Screening

Eligibility

Included
