# Supplementary material for: Association of common gene variants in glucokinase regulatory protein with cardiorenal disease: A systematic review and meta-analysis
Source: PLoS One. 2018 Oct 23;13(10):e0206174. doi: 10.1371/journal.pone.0206174 (PMC6198948; doi:10.1371/journal.pone.0206174)
Supplement: S3 Fig — (DOCX) [file pone.0206174.s009.docx]

**S3 Fig. Forest plot of the meta-analysis on CAD – sensitivity analysis**

**
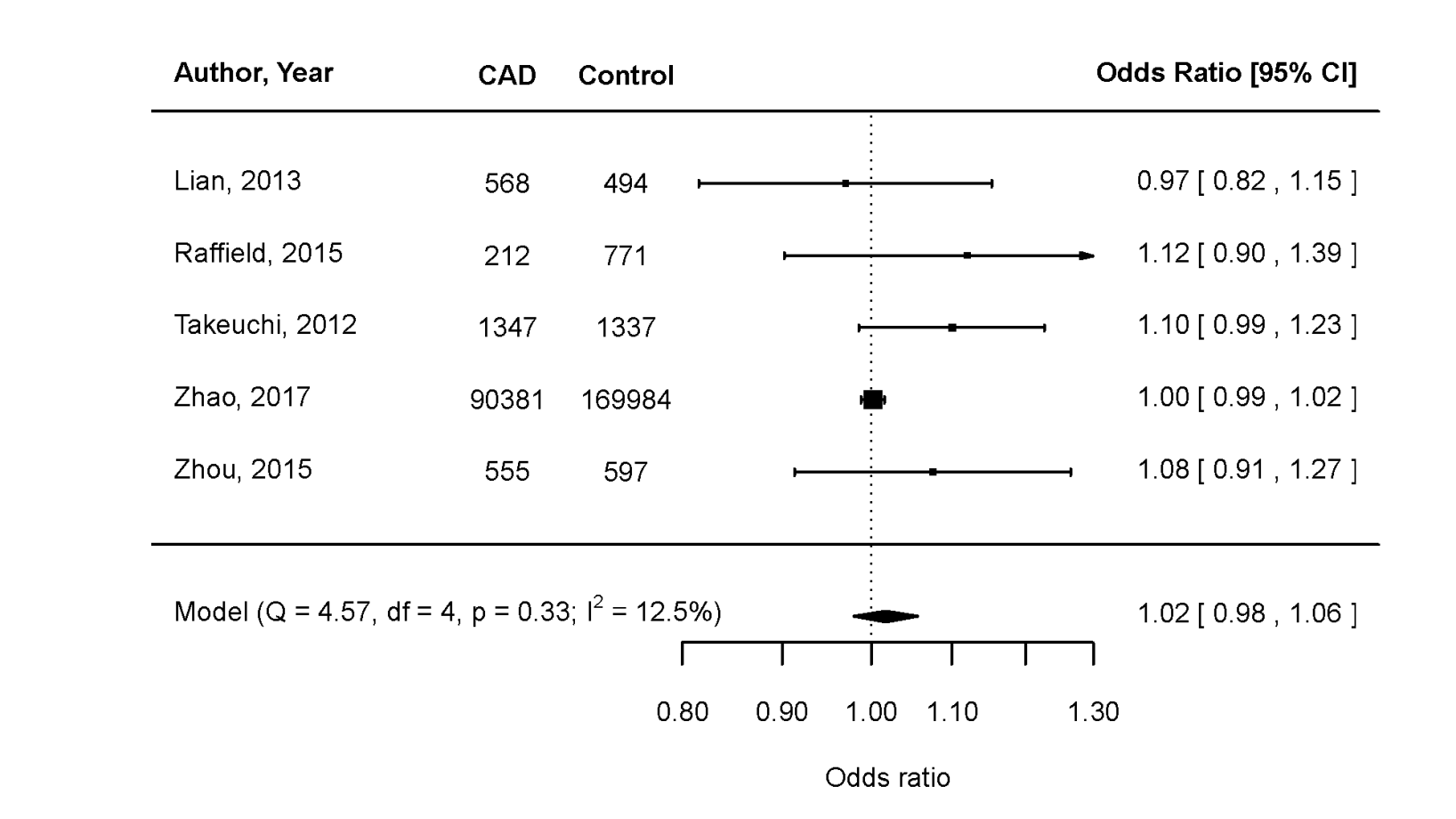
**

For this analysis, the combined UK Biobank, CARDIoGRAMplusC4D 1000 genomes-based GWAS, and Myocardial Infarction Genetics and CARDIoGRAM Exome dataset [1] was replaced by CARDIoGRAMplusC4D 1000 genomes-based GWAS dataset combined with 56,354 samples [2].

**References**

1. Nelson CP, Goel A, Butterworth AS, Kanoni S, Webb TR, Marouli E, et al. Association analyses based on false discovery rate implicate new loci for coronary artery disease. Nat Genet. 2017;49(9):1385-91. Epub 2017/07/18. doi: 10.1038/ng.3913. PubMed PMID: 28714975.

2. Zhao B, Lu Q, Cheng Y, Belcher JM, Siew ED, Leaf DE, et al. A Genome-wide association study to identify single-nucleotide polymorphisms for acute kidney injury. American Journal of Respiratory and Critical Care Medicine. 2017;195(4):482-90.
