# Supplementary material for: Association of common gene variants in glucokinase regulatory protein with cardiorenal disease: A systematic review and meta-analysis
Source: PLoS One. 2018 Oct 23;13(10):e0206174. doi: 10.1371/journal.pone.0206174 (PMC6198948; doi:10.1371/journal.pone.0206174)
Supplement: S4 Fig — (DOCX) [file pone.0206174.s010.docx]

**S4 Fig. Forest plot of the meta-analysis on CAD** – **stratified by ancestry**


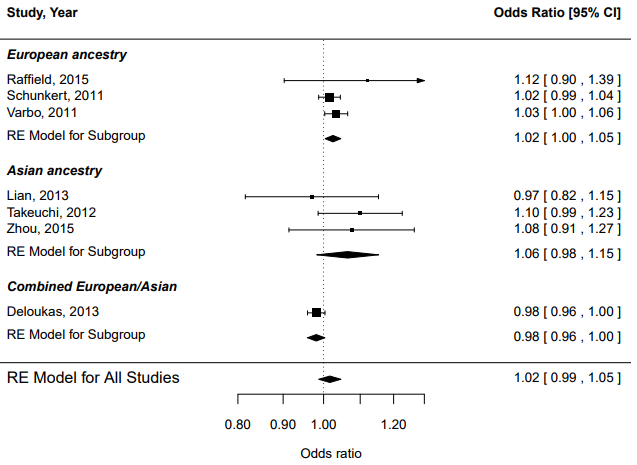


For this analysis, the combined UK Biobank, CARDIoGRAMplusC4D 1000 genomes-based GWAS, and Myocardial Infarction Genetics and CARDIoGRAM Exome dataset [1] was replaced by the CARDIoGRAMplusC4D Metabochip dataset [2, 3], which allows stratification by ancestry. The CARDIoGRAMplusC4D Metabochip dataset overlaps for ~55% with the CARDIoGRAMplusC4D 1000 genomes-based GWAS. The Copenhagen City Heart Study, the Copenhagen General Population Study, and the Copenhagen Ischemic Heart Disease Study [4] were not part of the CARDIoGRAMplusC4D Metabochip dataset and were therefore included in this meta-analysis.

**References**

1. Nelson CP, Goel A, Butterworth AS, Kanoni S, Webb TR, Marouli E, et al. Association analyses based on false discovery rate implicate new loci for coronary artery disease. Nat Genet. 2017;49(9):1385-91. Epub 2017/07/18. doi: 10.1038/ng.3913. PubMed PMID: 28714975.

2. Schunkert H, Konig IR, Kathiresan S, Reilly MP, Assimes TL, Holm H, et al. Large-scale association analysis identifies 13 new susceptibility loci for coronary artery disease. Nat Genet. 2011;43(4):333-8. Epub 2011/03/08. doi: 10.1038/ng.784. PubMed PMID: 21378990; PubMed Central PMCID: PMCPMC3119261.

3. Deloukas P, Kanoni S, Willenborg C, Farrall M, Assimes TL, Thompson JR, et al. Large-scale association analysis identifies new risk loci for coronary artery disease. Nat Genet. 2013;45(1):25-33. Epub 2012/12/04. doi: 10.1038/ng.2480. PubMed PMID: 23202125; PubMed Central PMCID: PMCPMC3679547.

4. Varbo A, Benn M, Tybjaerg-Hansen A, Grande P, Nordestgaard BG. TRIB1 and GCKR polymorphisms, lipid levels, and risk of ischemic heart disease in the general population. Arteriosclerosis, Thrombosis, and Vascular Biology. 2011;31(2):451-7.
