# Supplementary figures and images for: Association of common gene variants in glucokinase regulatory protein with cardiorenal disease: A systematic review and meta-analysis
Source: PLoS One. 2018 Oct 23;13(10):e0206174. doi: 10.1371/journal.pone.0206174 (PMC6198948; doi:10.1371/journal.pone.0206174)

**S5 Fig. Forest plot of the meta-analysis on CKD – stratified by ancestry**


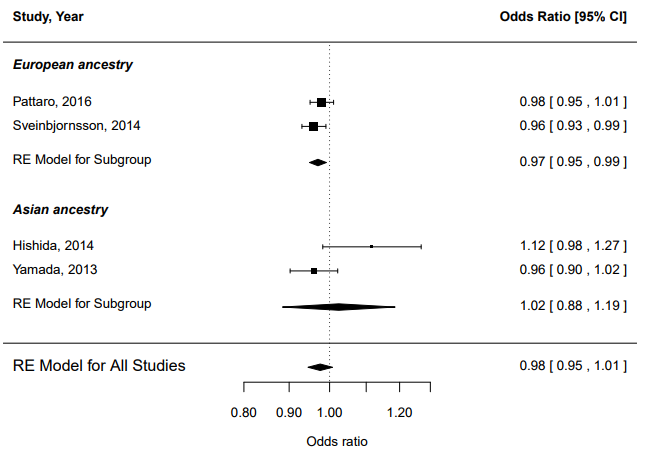

Supplement: S5 Fig — (DOCX) [file pone.0206174.s011.docx]
